# Teachers’ Beliefs and Instructional Implementation of Mathematical Problem Solving Within Project-Based Learning

**DOI:** 10.3390/bs16071091

**Published:** 2026-07-02

**Authors:** Yixuan Liu, Yiming Cao

**Affiliations:** 1 School of Mathematics and Statistics, Central China Normal University, No. 152 Luoyu Road, Wuhan 430079, China; yxliuccnu@ccnu.edu.cn; 2 School of Mathematical Sciences, Beijing Normal University, No. 19 Xinjiekouwai Street, Beijing 100875, China

**Keywords:** project-based learning, mathematical problem solving, teacher beliefs, mathematics curricula

## Abstract

In China’s latest mathematics curriculum, project-based learning (PBL) foregrounds mathematical problem solving (MPS) as student-led inquiry in authentic contexts. This study examines teachers’ beliefs about MPS within PBL, and how such beliefs are enacted in planning and teaching. Using a constant comparative approach, we drew on interviews, lesson plans, and reflective accounts from 14 mathematics teachers. Most teachers emphasised application and creativity, positioning themselves as guides/supporters who facilitate students’ exploration. Consistent with these beliefs, lesson plans commonly drew on personally and socially grounded contexts and featured challenging, open-ended tasks attending to cognitive breadth and depth. While espoused beliefs largely aligned with described implementation, a minority endorsed procedure-focused instruction, revealing tensions between exploration- and content-oriented approaches. Examining teachers’ beliefs can inform understanding of progress in curriculum reform. Sustained, long-term professional learning—supported by institutional resources and professional autonomy—is needed to help teachers navigate these tensions.

## 1. Introduction

“Not having heard something is not as good as having heard it; having heard it is not as good as having seen it; having seen it is not as good as knowing it; knowing it is not as good as putting it into practice.” (不闻不若闻之，闻之不若见之，见之不若知之，知之不若行之。).

Xunzi (1990, trans. Knoblock)

Traditional mathematics classroom teaching in China, as in most of East Asia, is dominated by teachers, with minimal student involvement (Leung, 2001). Thus, curriculum reforms in China have always focused on changes in teaching methods (Dello-Iacovo, 2009; Tan, 2015). China’s latest curriculum reform introduced project-based learning (PBL) in many disciplines, including mathematics, biology, chemistry, and history. The mathematics curriculum standard recommends PBL as a key approach to teaching and learning in the *zonghe yu shijian* (synthesis & practice) content area in upper primary and secondary stages (MOE China, 2022). The other three content areas are numbers & algebra, graphs & geometry, and statistics & probability. Mathematical problem solving (MPS) is embedded in every topic and grade in the mathematics curriculum (Lester & Cai, 2016), including PBL. However, within PBL, MPS is characterised by authenticity and challenge, central tasks that drive learning (Krajcik & Shin, 2014; Larmer et al., 2015). While some studies have demonstrated PBL’s effectiveness in enhancing students’ mathematical problem solving skills (e.g., Cujba & Pifarré, 2024; Rehman et al., 2023), others have found it to have little or no impact (e.g., Garner, 2021; Shchetynska, 2020). Nevertheless, teachers’ perspectives are crucial for determining why and how MPS is implemented within PBL.

Beliefs could be thought of as lenses that affect one’s worldview, or as dispositions towards action (Philipp, 2007). Teachers’ beliefs are critical for teaching practice, instructional implementation, and, more broadly, curriculum reform. Individually, teachers with different beliefs vary in teaching practices (Q. Zhang, 2022), yielding different students’ achievements (Y. Wang et al., 2022). Generally, teachers’ beliefs can help explain curriculum reform endeavours (Fullan, 2015; Handal & Herrington, 2003). Teachers’ beliefs about their roles, teaching objectives, and teaching strategies are critical to their teaching practices (Ernest, 1989a, 1989b; R. Li et al., 2024; Reichert et al., 2021). Thus, teachers’ beliefs can be regarded as pivotal in implementing curriculum and instruction.

As a new approach of teaching and learning, PBL requires emphasis on the role of teachers as curriculum developers. For example, Buck Institute for Education has established gold standard of essential project design elements for developing high-quality projects (Buck Institute for Education, 2019). Additionally, the curriculum standard also recommends teachers to design and develop appropriate projects (MOE China, 2022). As curriculum developers, teachers select and (re)design curriculum plans, as well as enact those plans in the classroom with students (Remillard, 1999). In addition, teachers also make decisions about the organisation, sequencing, and content of the curriculum as a whole (mapping arena in Remillard, 1999). Given the crucial role of MPS within PBL, teachers should also select, adapt and design MPS tasks and activities during the implementation of PBL.

Teachers’ beliefs are considered relevant to teaching practices. It has been revealed that both consistency and inconsistency can occur when considering individual teachers (Philipp, 2007; Q. Zhang, 2022). Facing inconsistency requires researchers’ more insights on teachers’ thinking about their context (Philipp, 2007). When a curriculum reform take place, teachers may change instructional beliefs to more constructional or insist more on traditional beliefs (Liljedahl, 2010). In China, the curriculum reforms follow a top-down model, and teachers have faced challenges and tensions during the transition in their teaching approaches (Law, 2014). In the context of designing and enacting PBL, this raises questions about whether teachers change their beliefs and/or their teaching practices regarding MPS, and whether such changes occur consistently.

Research questions are:

RQ1 What is the status of teachers’ beliefs on teachers’ role and teaching objectives and strategies regarding MPS within PBL after their instruction?

RQ2 How do teachers implement MPS instruction within PBL across the design, construction, and mapping arenas?

RQ3 How are teachers’ beliefs related to their instructional implementation of MPS within PBL?

## 2. Conceptual and Theoretical Framework

### 2.1. Mathematical Problem Solving Within Project-Based Learning

Project-based learning can be regarded as a contemporary development of the *project method* (Pecore, 2015). The project method was redefined and popularised by William H. Kilpatrick within modern progressive education (Kilpatrick, 1918), drawing on John Dewey’s concept of *learning by doing* (Dewey, 1938). However, PBL’s emphasis on authentic and practical learning resonates with order traditions, including apprenticeship, manual training in professional, technical, and art education, and Chinese pedagogical thought that values putting knowledge into practice (Billett, 2016; Xunzi, 1990, trans. Knoblock; Knoll, 1997). PBL is an inquiry-based instructional approach that reflects a learner-centred environment and concentrates on learners’ application of disciplinary concepts, tools, experiences, and technologies to research the answers to questions and solve real-world problems (Condliffe, 2017; Larmer et al., 2015). In recent years, PBL has been recommended in both Western and Eastern mathematics education systems (Lee & Galindo, 2021; MOE China, 2022; National Research Council, 2012). The effectiveness of PBL on mathematics achievement has been recognised by many studies (Boaler, 1998; Chen & Yang, 2019; D. W. Holmes, 2022; Jenkins, 2018; Rehman et al., 2023; Septian et al., 2020; Yancy, 2012).

Problems, especially mathematical problems, within PBL are typically authentic, challenging, open-ended and applicable (Buck Institute for Education, 2019; Lee & Galindo, 2021). On one hand, MPS can serve as a driving question that guides learners through the processes of the project, thereby fostering their learning of mathematical content knowledge (Krajcik & Shin, 2014; Lee & Galindo, 2021). On the other hand, MPS abilities are one of the key objectives of PBL (Lee & Galindo, 2021). PBL can complement classical MPS for students of varying ability levels, and enriching the learning environment (Halverscheid, 2005). However, some studies have found that integrating PBL into mathematics curriculum is challenging for both teachers and students: (a) tense of time (Cullen-Hamzeh, 2018) (b) teachers’ lack of confidence on mathematics content (Cullen-Hamzeh, 2018) (c) difficulties in integrating mathematical content into projects (Muir et al., 2016) etc. These factors can also influence teachers’ conceptions and practices of MPS within PBL.

### 2.2. Teachers’ Beliefs on Mathematical Problem Solving

Earlier research concentrated on teachers’ general beliefs about mathematics. For example, Ernest (1989a, 1989b) proposed three main components teachers’ beliefs regarding mathematics: the nature of mathematics, the teaching of mathematics and the learning of mathematics. However, the later research focus on specific topics in mathematics education, e.g., teaching through problem posing (X. Li et al., 2020), use of technology (Yang & Leung, 2015), understanding of curriculum standards (Zollman & Mason, 1992), and understanding of the gender difference in mathematics (Q. Li, 1999).

Theoretical frameworks focus on teachers’ beliefs about teaching MPS remain limited (Xenofontos & Andrews, 2014). One adapts a dichotomous classification of teachers’ beliefs about teaching MPS, distinguishing traditional and reformed orientations, corresponding to teacher- and student-centred teaching, respectively (Saadati et al., 2019). However, while MPS activities are often considered to be associated with reformed teaching practices and relative beliefs (Anderson, 2005; Handal & Herrington, 2003), e.g., teaching through problem-solving (Hourigan & Leavy, 2022), such a division of teaching approaches may not be applicable in Eastern instructional contexts, including China (Biggs & Watkins, 2001; Q. Zhang, 2022).

Another framework for understanding teachers’ beliefs about teaching MPS is grounded in their beliefs about the nature of mathematics. Safrudiannur and Rott (2021) developed this framework from data gathered on teachers’ beliefs about the nature of mathematics (Ernest, 1989a) and on teaching approaches (Beswick, 2005; Van Zoest et al., 1994). The framework integrates teachers’ roles as instructor, explainer or facilitator in terms of MPS, because they are likely to correspond to teachers’ beliefs (Berk & Cai, 2019).

*The Instrumentalist View—Content-Performance*. Mathematics is seen as a set of unrelated but utilitarian facts, rules, and skills (Ernest, 1989a). MPS requires remembering knowledge as well as the calculation and reasoning process. A teacher in this category directs students toward a proposed problem-solving method that students are expected to follow, memorise, and recall (Safrudiannur & Rott, 2021). This teacher is an *instructor*.

*The Platonist View—Content-Understanding*. Mathematics is seen as a static, unified body of knowledge to be discovered, not created (Ernest, 1989a). MPS requires understanding and mastering knowledge, as well as the calculation and reasoning process. A teacher in this category begins by modelling one or more solution methods, allows students to choose one, and provides suggestions and explanations to help students when they reach an impasse (Safrudiannur & Rott, 2021). This teacher is an *explainer*.

*The Problem-Solving View—Learner-Interaction*. Mathematics is seen as a dynamic, continually expanding field of human creation and invention, a cultural product (Ernest, 1989a). MPS requires students to explore and generate both reasoning and outcomes. A teacher in this category provides only the information and guidance necessary for students to create and follow their own solution methods (Safrudiannur & Rott, 2021). This teacher is a *facilitator*.

This categorical approach is adopted in this study because it appears to offer a stronger explanatory power for understanding practices for teaching problem solving (Rott, 2020; Q. Zhang, 2022). In addition, as learning objectives and their attainment are essential in PBL (Larmer et al., 2015; MOE China, 2022), it incorporates teachers’ beliefs regarding learning MPS objectives within PBL.

### 2.3. Teachers as Curriculum Designers

Per China’s Ministry of Education (MOE China, 2022, p. 78), “The central principle of project-based learning is the identification of appropriately designed projects.” Adopting the novel instructional approach of PBL requires teachers to engage in a cyclical process of training, (re)design, enactment, and reflection (Larmer et al., 2015). Even when PBL teaching materials are provided, teachers must adapt them for their instructional implementation, highlighting the teacher’s role as curriculum designer when engaged in curriculum reform (Remillard, 1999).

Remillard’s (1999) theoretical framework for teachers as curriculum developers is adapted for this study. Three arenas—design, construction, and mapping—are employed to describe the curriculum development stages in which teachers make selections and decisions about MPS within PBL. In the design arena, teachers select and design mathematical problems. In the construction arena, teachers enact these mathematical problems and respond to students’ encounters with them in classrooms, with both actions being either design-based or improvised. Finally, in the mapping arena, teachers make choices that determine the organisation and content of the curriculum, particularly regarding MPS within PBL. Although this arena is not directly related to teaching practices, it impacts and is impacted by them. In addition, decisions in this area set the context for the activities of the other two arenas. The three-arena model, adapted with a specific focus on MPS within PBL, is employed in this study to describe and analyse teachers’ instructional implementation.

## 3. Methods

### 3.1. Participants

Purposive sampling was used in this study. Participants were selected based on the following criteria: (a) they were experienced mathematics teachers; (b) they had experience in designing and enacting PBL in classroom teaching; (c) they were willing to participate in this study and share their experience; and (d) variation in gender, years of teaching experience, and geographical region was considered to capture diverse perspectives (see Table 1). For convenience, two of the 14 participants, T5 and T10, were selected to provide video recordings of their classroom teaching.

### 3.2. Data Collection

This study mainly collected interview data from teachers along with their lesson designs. All lessons were designed, enacted, and reflected upon by the participating teachers and their colleagues, highlighting the teachers’ role as curriculum designers. The interviews highlighted teacher reflection, a key factor in changing teacher beliefs and practices (Philipp, 2007).

Interview data included teachers’ backgrounds (experience, profile and students’ abilities) and their beliefs about and instructional implementations of MPS within PBL. In terms of beliefs, teachers were asked to compare how they taught MPS within PBL and MPS within their daily classrooms, focusing on their teaching objectives, roles, and strategies. Regarding strategies, three statements adopted from Safrudiannur and Rott’s (2021) questionnaire were provided, each representing a different view. Teachers were allowed to express their endorsement of one or more views. Teachers were also asked to explain their related teaching beliefs.

Regarding the instructional implementation of PBL, this study employs the three-arena model. In the interviews, teachers were asked to explain, in general terms, their lesson-design process and then to describe their decision-making process when designing and enacting MPS tasks (in the design arena). Teachers’ considerations in selecting themes and content were also included in interviews (in the mapping arena). Teachers were also asked to describe how PBL was positioned within the mathematics curriculum, as well as its affordances and constrains in relation to MPS (in the mapping arena).

In addition to interviews and lesson designs, two teachers, T5 and T10, provided video recordings of one classroom lesson each, lasting approximately 40 min (in the construction arena). Both lessons took place in a recording classroom, with 30 students participating in each lesson. Their interviews also covered the strategies they used to guide MPS in the videotaped lessons. Overall, the data collected primarily concerned instructional implementation in the design and mapping arenas, as in previous studies (McFadden & Roehrig, 2017; Wallace & Priestley, 2011).

### 3.3. Data Analysis

As PBL and MPS were new to the mathematics curriculum, the *constant comparative method* was adopted in this study to investigate and generate categories of teachers’ beliefs and instructional implementations in the design and mapping arenas. This method usually involves four analytic stages: (a) comparing incidents applicable to each category; (b) integrating categories and their properties; (c) delimiting the theory; and (d) writing theory (Glaser, 1965). In the process of data analysis, teachers’ perspectives were first divided into different domains. Relevant categories, such as teachers’ role as guides, were generated through the coding of teachers’ statements. The initially coded categories were then compared and further integrated based on their meanings. Finally, relationships were established both within and across categories concerning teachers’ beliefs and instructional implementations.

For the video data, a case study approach was adopted. The two recorded lessons provided by T5 and T10 were treated as illustrative cases of the construction arena and were analysed alongside the corresponding interviews and lesson designs. The analysis focused on how teachers enacted teachers’ roles and teaching strategies during MPS within PBL, particularly how they facilitated students’ problem-solving processes. The video data therefore complemented the interview and lesson design data by showing how teachers’ beliefs were reflected in teaching practices (in the construction arena).

This study mainly gathered textual data. The interviews were recorded with the interviewees’ permission, transcribed using transcription software, and subsequently manually proofread for accuracy. MAXQDA2020 was used to code and further analyse the data.

## 4. Findings

### 4.1. Teachers’ Beliefs Toward the Learner-Interaction Perspective

Almost all participating teachers described MPS in daily classroom teaching as emphasising teacher exposition, close adherence to predetermined instructional plans, and largely passive student participation in content mastery and understanding.

First, regarding the MPS teaching objectives within PBL, most teachers (10 out of 14) agreed on applying knowledge and fostering creativity. They believed MPS within PBL offered students the opportunity to apply knowledge in real-life contexts and thereby realise the practical value of mathematics. Additionally, 9 of the 14 teachers identified learning interest and 8 identified motivation as key teaching objectives. For example, T7 said, “Interest is the best teacher … If I can spark even a tiny bit of interest in them through these [problem-solving activities], it will be immensely rewarding.” Elsewhere, teachers mentioned specific aspects of teaching objectives, such as data sense, modelling sense, reasoning ability, and abstract thinking ability. However, some teachers felt that certain students lacked opportunities to express themselves mathematically in daily classroom settings, making collaboration during MPS within PBL difficult. Some teachers also agreed that students weak in mathematical knowledge had more difficulty participating in MPS within PBL.

After merged the initial descriptions of teachers’ roles, the results show that most of the 14 teachers concurred that they served as guides (9) or supporters (7) of students in MPS within PBL (see Table 2). Guiding teachers led the problem-solving process using structured methods or strategies, emphasising their role in scaffolding students’ investigations. As T6 said,

At first, we gave them (students) quite a lot of freedom. But during the task, we found that some students did not know how to start when choosing statistical measures, partly because of their ability level. So later on, we still had to give them a rough framework, so that they knew what to do. Otherwise, things could get a bit out of control, or even go off topic and lose the mathematical focus.

In addition, T3 said, “We need to keep guiding students towards the final goal.” This perspective emphasises teachers’ scaffolding in guiding students in the directions of MPS objectives. Supporting teachers provided assistance only when necessary, allowing students to explore the problem-solving process and generate related ideas independently. As T5 noted, students should be allowed to “generate it themselves”, and T8 suggested that students’ responses should “drive the lesson forward”. T2 further highlighted this supportive role:

When carrying out any project, I think students should be allowed to go as far as they can. The teacher’s role is more about support, or providing them with some help. It should not start from the teacher’s perspective… If students can reach, or even go beyond, the goal with a little prompting from me, that would be the best. If not, I would not force them to achieve what I want, but would give them more room to develop.

T7 also described the similar perspective. Despite emphasising different aspects of teaching, these two roles were not mutually exclusive, and some teachers integrated both into their teaching beliefs and practices—for example, guiding students to understand the problem first and then encouraging them to investigate autonomously, providing support only when needed. However, two teachers identified with the teacher’s role as an instructor, emphasising direct intervention in students’ problem-solving outcomes. By comparison, teachers’ roles as organisers (3), assessors (3) and designers (1) appear less prominent, as these roles are more closely associated with the general implementation of PBL rather than the specific process of MPS.

Regarding MPS teaching strategies, based on the categorical approach adopted in this study, most teachers (13) endorsed the learner-interaction view, with about two-thirds (8) agreeing with it fully. The learner-interaction view emphasises encouraging students to explore autonomously during MPS, without teacher-provided clues. For example, T8 said:

Project-based learning is primarily intended to help students develop the ability to think independently and to work together to solve problems. So, if we give them certain clues or scaffolds, won’t that steer their thinking toward a (preset) direction? … After all, PBL also aims to help students think like scientists, and that means many problems are supposed to be unknown. If we provide ready-made ideas, it might get in the way of their collaboration and discussion.

T5 shared her beliefs concerning MPS within PBL versus within daily classrooms:

In daily classrooms, we usually guide students to follow one set path (to solve the problem). But project-based learning is much more open. Some students really do come up with ideas that start in one direction and lead to others, and that’s fine—I just follow their directions, and many situations go beyond anything we expected. So, I think that’s the most significant difference [between MPS within PBL and within daily classrooms].

About one-third of the teachers (4) supported integrating the learner-interaction view with the content-understanding view, highlighting students’ autonomy in exploration and their understanding of the relative knowledge, techniques, and strategies required for mathematical problem-solving. Two teachers, T4 and T7, agreed that teachers should first allow students to engage in exploration, then provide them with scaffolding to strengthen their understanding. As T7 noted,

First, I don’t give them any clues. I just ask students, ‘How would you like to solve this?’ After (s)he write down their ideas, I’ll try to make sense of what they’ve done, and then we’ll talk it through together. We look at how they used (the knowledge or skills) when problem solving—whether they used them well, what worked, what could be improved, and where they might adjust their approach. … So, I start by letting students think freely without restricting them. Then we narrow down the problem a bit, … and finally we work on refining their solutions.

The other two teachers, T1 and T2, believed that providing clues first is necessary to help students understand the problem and then independently investigate possible solutions. Only one teacher, T10, supported integrating the learner-interaction view with the content-performance view. She agreed that students should explore first, with teachers providing direct instruction only when they make mistakes or are unable to solve problems, to help them master the material and correct their errors.

In addition, only one teacher, T6, supported the content-understanding view fully. She believed content-understanding should occur throughout the MPS process. Students need to understand what is required first, then the problem-solving process, highlighting the importance of continuous guidance and reflection during learning.

### 4.2. Promoting Challenging and Authentic MPS During Instructional Implementation

The three arna model is adopted in this study for analysing instructional implementation. As curriculum developers, the participating teachers implemented their PBL lessons by designing mathematical problems and their contexts, selecting topics, arranging the content, and enacting the lessons in their classrooms. The focus of this study, in terms of instructional implementation, is on the design and mapping arenas. Only two teachers’ lessons were observed concerning the construction arena. Each PBL in this study included only one core MPS task, which was then analysed (see Supplementary Materials).

In the design arena, participating teachers set up various contexts for MPS. MPS’s cognitive demands were analysed using Smith and Stein’s (1998) framework, and most (10 of 14) were classified as *doing mathematics*. These problems involved complex mathematical thinking and reasoning, in which students posed and tested hypotheses, broke down and formulated subproblems, and found paths to solutions. Additionally, half of the problems were open-ended, allowing multiple methods and leading to multiple solutions. However, two teachers, T1 and T9, designed low-cognitive-demand MPS that required applying procedures with little connection to underlying mathematical concepts and meanings (see Supplementary Materials). Based on the contexts outlined in the PISA 2022 mathematics framework (OECD, 2018), the mathematical problems in this study were approximately equally distributed amongst four contexts: personal (3), occupational (4), societal (3), and scientific (4).

In the construction arena, T5 and T10 both encouraged students’ investigations and mathematical representations in their classrooms, enacting their student-centred perspective by encouraging the use of various methods, valuing students’ approaches, and enabling them to fully express their views when disagreements arose. In addition, both teachers’ instruction was intended to facilitate the exploration and generation of new mathematical knowledge. Based on the interview data, teachers’ guidance was improvised, with little consideration given to facilitating exploration. Their emphasis on standardising mathematical language constrained students’ explorations somewhat, leading them to pursue more “standardised” solutions that aligned with their teacher’s expectations, contradicting the very nature of autonomous inquiry. For example, T5 expected students to provide answers in standard mathematical language; although she was happy when students expressed their answers in their own words, she would eventually provide the standard answer, without incorporating their contributions. As for T10, although she encouraged students to solve problems on their own, she checked their answers throughout and provided direct instruction while guiding them. In the follow-up interview, after watching her classroom video recording, T10 recognised a mismatch between her current instructional practices and the facilitative role expected in PBL, and admitted needing professional support to bridge the gap between ideal and actual classroom teaching practices.

In the mapping arena, topic selection and content organisation aim to ensure that MPS is challenging but grounded in familiar themes. Participating teachers tended to select topics connected to their personal experiences and societal backgrounds—social background (five), personal experience (four), textbooks (four), and exercises (two)—and then formulate appropriate mathematical problems. Despite the diversity of sources, most topics foregrounded authenticity. In terms of the content organisation, the participating teachers consistently designed mathematical problems that incorporated substantial mathematical concepts and knowledge.

For example, T2 designed a problem that involved investigating how to minimise material usage for a 250 mL milk carton (given that the length-to-height ratio was the golden ratio). The problem integrated mathematical content from different areas and stages, including nets, surface area, the volume of a cuboid, and the concept and application of functions. These problems involved making internal connections within mathematical content, were challenging, and required MPS within PBL that focused on transformation—i.e., transferring mathematical knowledge and skills from familiar to unfamiliar situations.

**Figure 1:**
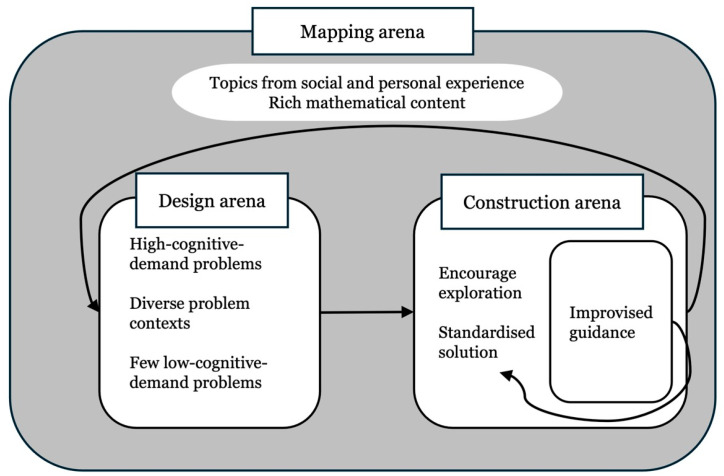
Prevalent patterns of instructional implementation across three arenas.

Overall, instructional implementation across the three arenas was not isolated but appeared to be interconnected (see Figure 1). Within the mapping arena, teachers established the decision-making background for the design and construction arenas and enacted instructional strategies in both. Most teachers selected topics from their social backgrounds and personal experiences, integrating rich connected mathematical content (mapping arena). Correspondingly, most problems involved high cognitive demands and were set in authentic contexts (design arena). Similarly, in the observed lessons, both teachers encouraged students to express their own thoughts and engage in independent exploration. In addition, teachers’ in-the-moment responses during classroom instruction tended to be improvised, which led to an emphasis on standardised solutions (construction arena). The teachers’ design and enactment reflected interactional alignment. Some of the teachers reported that they refined their lesson plans based on their teaching practice. In addition, T5 and T10 stated that they would revise their lesson plans based on their reflections on teaching practice. However, misalignment also occurred in, for example, comprehensive topics that included low-cognitive-demand problems within social contexts (see T1 in Supplementary Materials).

### 4.3. Consistency and Inconsistency Between Teachers’ Beliefs and Instructional Implementation

Teachers’ beliefs about and instructional implementation of MPS within PBL displayed both consistency and inconsistency. Most participating teachers consistently designed challenging mathematical problems that featured cognitive breadth and depth, reflecting their beliefs that MPS within PBL could foster the application of mathematics and creativity. They also agreed with the idea of teachers acting as guides and supporters, and endorsed the learner-interaction and content-understanding views, which were consistent with their design of problems intended to promote exploration and the integration of mathematical concepts. Most problems were authentic and set in social contexts and personal experiences, which was also consistent with their agreement on the application of knowledge. The two teachers whose lessons were observed primarily encouraged students to engage with the problems and express their ideas, consistent with their endorsement of the learner-interaction view. Nevertheless, this consistency was not limited to reform-oriented views. T9’s endorsement of the teacher’s role as an instructor was consistent with his design of the low-cognitive-demand problem. T10 also endorsed the content-performance view, consistent with her providing direct instruction during guidance.

However, inconsistency was also observed during this study. T1 endorsed the learner-interaction view but designed low-cognitive-demand, closed-ended problems. In addition, T5 and T10 both focused on “standardised” results when offering guidance in their observed lessons, inconsistent with their learner-interaction view. T10 represented a particularly complex case, as her beliefs and instructional implementations combined both traditional and reform-oriented elements. This mixed pattern made the relationship between beliefs and instruction less straightforward than in the other cases. Nevertheless, despite some inconsistencies, consistency was more prevalent across all the cases.

## 5. Discussion

In response to RQ 1, regarding the cognitive objectives, the participating teachers believed that applying knowledge and fostering creativity were key objectives of MPS within PBL, echoing current curriculum standards (MOE China, 2022). Regarding the non-cognitive objectives, the participating teachers believed that MPS within PBL can enhance students’ motivation and learning interest in mathematics, which has been confirmed (V.-L. Holmes & Hwang, 2016; Worry, 2011). Most participating teachers believed they were supporters (embracing the learner-interaction view) and/or guides (embracing the content-understanding view). The supporters in this study were akin to what Ernest (1989a) called facilitators—teachers who provide support only when needed and allow students maximum scope for autonomous exploration. The guides in this study were similar to what Ernest (1989a) called explainers—teachers who lead students through one or more fixed, structured solution methods, with an emphasis on conceptual and/or procedural understanding. These teachers’ roles and views on teaching MPS also aligned with the curriculum standards’ emphasis on students’ autonomous inquiry (MOE China, 2022), as well as the relevant principles of PBL (Barron et al., 1998; Buck Institute for Education, 2019; Helle et al., 2006; Krajcik & Shin, 2014). Nevertheless, a very small minority of teachers, embracing the content-performance view, endorsed their role as instructors and focused solely on mastering methods. As PBL is a new teaching and learning modality, such beliefs may stem from teachers’ daily classrooms, which are similar to those found in traditional East Asian learning contexts (Leung, 2001). Teachers’ beliefs in this study represented an eclectic synthesis of established and emerging beliefs regarding teachers’ roles and teaching strategies, extending the findings reported by Zheng (2013).

In response to RQ2, the findings suggest that teachers’ instructional implementation varied across the design, construction, and mapping arenas, reflecting the distinct pedagogical demands of each arena. In the design arena, teachers in this study implemented MPS within PBL by fostering students’ investigation of challenging problems that featured cognitive depth and breadth and were situated in familiar authentic contexts and themes. The dominant category of cognitive demands was *doing mathematics*—i.e., requiring students to access relevant knowledge and skills autonomously and use them appropriately in problem-solving through exploration and complex thinking (Smith & Stein, 1998). This trend may be considered a defining feature of the reform (e.g., Ubuz et al., 2010). Contextually, the problems were relatively evenly distributed, reflecting the concept of situated learning (Krajcik & Shin, 2014), PBL’s authenticity principles (Buck Institute for Education, 2019; Grossman et al., 2019; Krajcik & Shin, 2014), and teaching practices for border disciplines (Pupik Dean et al., 2023).

In terms of teaching practices (construction arena), the participating teachers encouraged students’ investigations, aligning with PBL’s principle of autonomous student inquiry (Barron et al., 1998; Buck Institute for Education, 2019; Helle et al., 2006; Krajcik & Shin, 2014). However, they tended to focus on “standard” results, as they often do in their daily classrooms, a tendency that resembles practices commonly seen in traditional Chinese mathematics classrooms (Cai, 2004; Mok, 2006). In addition, teachers tended to check students’ solutions, and students were inclined to seek teachers’ confirmation, echoing S. Zhang et al. (2022). Thus, teachers included daily classroom teaching as part of their implementation of MPS within PBL. In this study, classroom observations and interviews suggested that teachers’ guidance was often improvised and insufficiently oriented toward supporting exploration. This appeared to direct students’ attention toward obtaining expected solutions rather than engaging in the problem-solving process, which may have undermined their active investigative engagement (Hand et al., 2013).

Regarding the mapping arena, the participating teachers integrated well-connected, wide-ranging mathematical content with themes informed by their experience, derived from daily life, social media, and classroom materials such as textbooks or exercises. These efforts reflect more demanding requirements for MPS and have been shown to support students’ mathematical thinking and reasoning (Jonsson et al., 2014; Stein et al., 1996). The mapping arena (Remillard, 1999), where teachers designed and enacted challenging and authentic problems, provided a decision-making background for the other two arenas through its authentic topics and enriched mathematical content, which influenced teachers’ design and practice.

The relationship between teachers’ beliefs and teaching implementation showed both consistency and inconsistency (RQ3), as reported in previous studies (Philipp, 2007; Q. Zhang, 2022). However, unlike most previous studies, this study expanded the scope of teachers’ practices across three arenas to examine those consistencies and inconsistencies. The observed consistencies indicated that teachers not only endorsed but also enacted MPS teaching in ways that aligned with established expectations (Lester & Cai, 2016), characterised by student-centred, exploration-oriented, and contextualised problem-solving approaches, with approximately half of the problems open-ended. However, the observed inconsistencies indicated that teachers’ beliefs were not fully realised in either instructional design or enactment. Such inconsistencies could be illustrated by the distinction between the ideal (espoused) and real (practical) aspects of teaching (Liljedahl, 2009). This gap is not limited to general mathematics teaching; it has also been documented in previous PBL practices (Cullen-Hamzeh, 2018; Muir et al., 2016). This separation warrants careful attention, as it may undermine the implementation of curriculum reform. Addressing this issue requires honest consultation, critical self-reflection, and improved communication within teachers’ organisations (Handal & Herrington, 2003).

Overall, consistencies were predominant in this study, while inconsistencies were relatively few. This suggests that PBL, as a reform initiative, was largely effective in supporting MPS implementation among the teachers in this study. The reason may lie in teachers’ core (control) beliefs (Tan, 2015). The teachers in this study did not view the reform as an abrupt change but rather as a further, incremental development based on prior practices. Teachers mentioned many principles that originated from previous mathematics curriculum reforms, including student-centredness, cultural values, attitudes towards mathematics, and the appreciation of mathematics (L. Wang et al., 2017).

## 6. Conclusions and Outlooks

This study demonstrates how PBL—a key component of mathematics curriculum reform in China—relates to teachers’ beliefs and is implemented in classrooms, with a specific focus on MPS. Most teachers endorsed a student-centred, exploration-oriented, contextualised approach to teaching MPS within PBL (RQ1). They generally enacted these beliefs by integrating authentic contexts, familiar themes, and MPS tasks that required broad and deep cognitive engagement (RQ2). Although no causal relationship was established, the consistency between teachers’ beliefs and instructional implementation indicates that the idea of PBL has been recognised and practised by most of the participating teachers. Although relatively few, the observed inconsistencies and perspectival biases revealed tensions between teachers’ espoused ideals and practical approaches, as well as between exploration-oriented and content-oriented approaches (RQ3). These tensions suggest that classroom curriculum reform is unlikely to be achieved overnight and may require sustained, long-term efforts, with continued professional support to help teachers navigate them in practice, as T10 mentioned. Although this study was situated in the Chinese context, it contributes to the broader international literature on MPS, PBL, and teachers’ enactment of curriculum reform. Overall, these results suggest that teaching MPS within PBL broadly aligns with policymakers’ intentions and is largely achievable in classroom practice. These findings also indicate that investigating teachers’ beliefs can provide useful insights into how widely and deeply curriculum reform has permeated classroom practice.

Regarding MPS within PBL, the participating teachers believed that instruction should be oriented towards “teaching *for* problem solving” (Schroeder & Lester, 1989), with particular attention to students’ ability to transfer what they have learnt from familiar problems to unfamiliar ones. The MPS tasks in this study presented more integrated content and more authentic contexts. On the one hand, PBL provides an open learning environment for MPS, complementing traditional MPS; on the other hand, PBL could lead to less mathematical content than planned (Halverscheid, 2005). In this study, both scenarios were observed by the researchers and reported by the teachers, reflecting the affordances and constraints of PBL. In curriculum terms, PBL may be better placed to complement—rather than replace—traditional mathematical problem solving.

As Handal and Herrington (2003) stated, “Successful curriculum change is more likely to occur when the curricular reform goals relating to teachers’ practice take account of teachers’ beliefs.” This study found that teacher belief is a prerequisite for implementing reforms; in other words, belief must come first—without it, teaching reform is unlikely. Teacher agency also played a crucial role in this study. Echoing previous research (Wallace & Priestley, 2011), changes in classroom practices were possible when teachers’ beliefs aligned with the reform philosophy and when teachers were empowered to develop reform methods in their classrooms. Accordingly, this study examined curriculum reform as designed and enacted by teachers within an enabling context that afforded professional autonomy and institutional support. Similar reforms are also facilitated by enabling contexts that support teacher agency.

This study has several limitations that should be acknowledged. First, as this study employed purposive sampling, teachers who had no experience with or interest in PBL were not invited to participate. However, their perspectives and reasons for resistance remain important and should be explored in future studies. Second, the findings are mainly based on interview data and lesson-design materials, with limited data derived from classroom observations. Third, this research was conducted during the implementation phase of curriculum reform, i.e., “the first experiences of attempting to put an idea or reform into practice” (Fullan, 2015, p. 65), which may limit the generalisability of the findings. Teachers’ beliefs and practices during other phases, especially the institutionalisation phase—in which changes are either embedded as an ongoing part of the system or are attenuated in (or even absent from) decision-making—warrant further attention in future research.

## Figures and Tables

**Table 1:** Participants’ profile.

Teacher	Gender	Exp. (Yr.)	Province
T1	F	21	Henan
T2	F	7	Zhejiang
T3	F	9	Beijing
T4	M	2	Beijing
T5	F	4	Beijing
T6	F	8	Henan
T7	F	16	Guangdong
T8	F	6	Zhejiang
T9	M	14	Guangdong
T10	F	3	Liaoning
T11	F	10	Beijing
T12	F	30	Heilongjiang
T13	F	15	Beijing
T14	F	5	Beijing

**Table 2:** Teachers’ roles and relevant descriptions.

Merged Roles	Initial Roles	Descriptions
Guides (9)	Guides (9)	Teachers guide students towards the predetermined learning objectives by providing scaffolding.
Supporters (7)	Supporters (5) Assistants (1) Helpers (1)	Students mainly generate outcomes independently to advance the lesson, while teachers provide support rather than pursue teacher-defined objectives.
Organisers (3)	Organisers (3)	Teachers organise classroom activities and encourage students to participate in PBL activities.
Assessors (3)	Assessors (3)	Teachers review and evaluate students’ PBL outcomes
Instructors (2)	Instructors (1) Controllers (1)	Teachers control the teaching process and instruct students in understanding and solving problems.
Designers (1)	Designers (1)	Teachers design and plan activities purposefully.

## Data Availability

The data, except for the video recordings, are available from the corresponding author on reasonable request. The video recordings are not publicly available due to privacy restrictions.

## Supplementary Materials

The following supporting information can be downloaded at: https://www.mdpi.com/article/10.3390/bs16071091/s1.

## Abbreviations

The following abbreviations are used in this manuscript:

PBL	Project-based Learning
MPS	Mathematical Problem Solving
