# Supplementary material for: Teachers’ Beliefs and Instructional Implementation of Mathematical Problem Solving Within Project-Based Learning
Source: Behav Sci (Basel). 2026 Jul 2;16(7):1091. doi: 10.3390/bs16071091 (PMC13404792; doi:10.3390/bs16071091)
Supplement: Supplementary file 1 [file behavsci-16-01091-s001.zip › behavsci-4289266-supplementary.pdf]

Teacher	Teachers' role	Teachers' Beliefs	Mathematical problems	Cognitive demands	Open/Close-ended	Context	Sources of topics	Mathematical content
T1	Designer, organiser and guide	Learner-interaction & Content-understanding	If each student consumes 400 grams of rice per day, how many kilograms of rice would the entire class (assuming 50 students) consume per day?<br><br>如果每个同学每天消耗 400 克大米，那么全班同学（按 50 人计算）每天大约浪费多少千克大米？	Procedures without connections	Close-ended	Personal	Social background<br><br>Word food day.	Multiplication Operations; Averages
T2	Guide, organiser and supporter	Learner-interaction & Content-understanding	Investigating the problem of minimising material usage for a 250ml milk carton (given that the ratio of length to height is the golden ratio).<br><br>探究 250 毫升的牛奶包装盒的最省用料问题（已知长和高的比例是黄金分割）	Doing mathematics	Close-ended	Societal	Textbook	Function; surface area and volume of a rectangular prism
T3	Guide	Learner-interaction	How can an exit wayfinding (signage) system be designed in the Temple of Heaven Park?<br><br>在天坛公园中如何设计出口导引方案？	Doing mathematics	Close-ended	Societal	Social background<br><br>Exit of the Temple of Heaven Park	Perpendicular bisector; measuring

			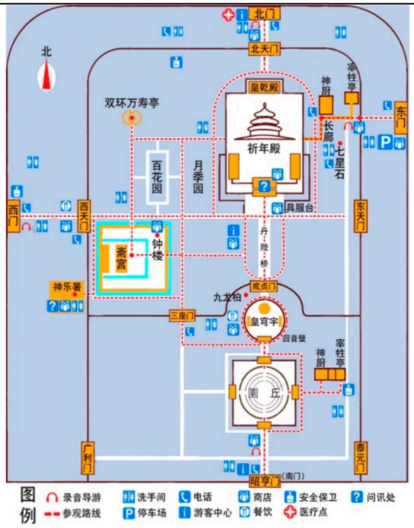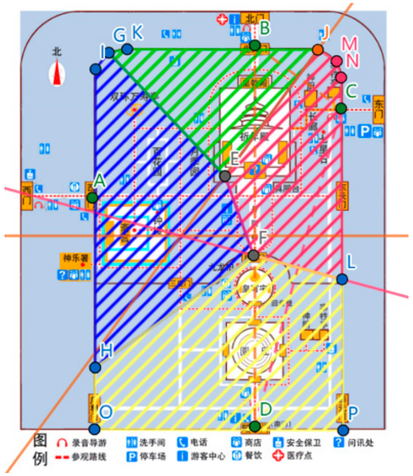					
T4	Guide and supporter	Learner-interaction & Content-understanding	For a 4×4 grid, how can you represent a four-digit number using black and white (filled and blank) squares?<br><br>对于一个 4×4 的格子，如何用涂黑和留白来表示一	Doing mathematics	Open-ended	Scientific	Exercise	Permutations and combinations

			个 4 位数？<br>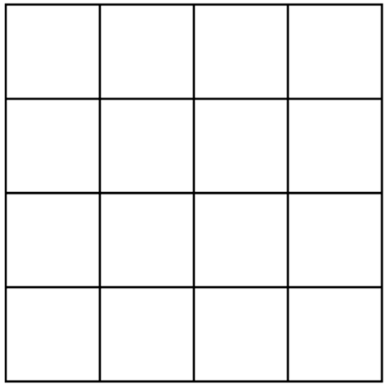					
T5	Supporter	Learner-interaction	Investigating how post-fold cutting methods affect the resulting five-pointed star (step 11).<br>探究折纸后裁剪方式与所得“五角星”的关系。<br>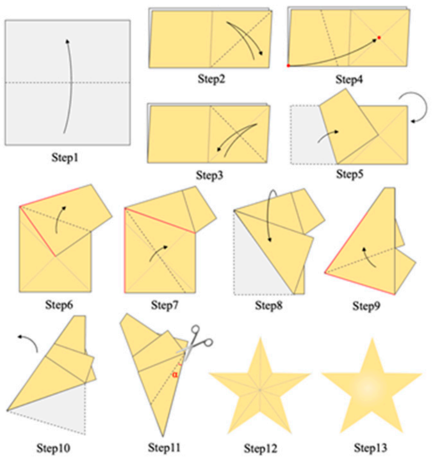 	Doing mathematics	Open-ended	Scientific	Social background<br><br>From social media	Symmetry and pentagram
T6	Guide	Content-understanding	Using your existing statistics knowledge, choose appropriate statistical measures to describe the bean sprout growth in different groups.	Doing mathematics	Open-ended	Occupational	Textbook	Mean, median, mode, and statistical charts

<table>
<tr><td></td><td></td><td></td><td>用已有统计学知识，选择合适统计量来描述不同组的豆芽生长情况。</td><td></td><td></td><td></td><td></td><td></td></tr>
<tr><td>T7</td><td>Guide, supporter, designer, organiser, and assessor</td><td>Learner-interaction & Content-understanding</td><td>Given a sequence of watering dates for certain plants, find the pattern of the sequence.
已知一些植物浇水日期，求解其中的规律。
<table>
<tr><th>Sunday</th><th>Monday</th><th>Tuesday</th><th>Wednesday</th><th>Thursday</th><th>Friday</th><th>Saturday</th></tr>
<tr><td></td><td></td><td></td><td></td><td></td><td></td><td>1</td></tr>
<tr><td>2</td><td>3</td><td>4</td><td>5</td><td>6</td><td>7</td><td>8</td></tr>
<tr><td>9</td><td>10</td><td>11</td><td>12</td><td>13</td><td>14</td><td>15</td></tr>
<tr><td>16</td><td>17</td><td>18</td><td>19</td><td>20</td><td>21</td><td>22</td></tr>
<tr><td>23</td><td>24</td><td>25</td><td>26</td><td>27</td><td>28</td><td>29</td></tr>
<tr><td>30</td><td>31</td><td>1</td><td>2</td><td>3</td><td>4</td><td>5</td></tr>
</table>
</td><td>Procedures with connections</td><td>Close-ended</td><td>Occupational</td><td>Exercise</td><td>Basic arithmetic operations</td></tr>
<tr><td>T8</td><td>Supporter and assessor</td><td>Learner-interaction</td><td>Throw a solid rubber ball: based on the graph of the measured horizontal and vertical distances, construct a function graph to describe the relationship between position (coordinates).

投掷实心球，根据所得的水平距离和垂直距离图表，建立函数图形来描述位置关系。</td><td>Doing mathematics</td><td>Close-ended</td><td>Personal</td><td>Personal experience

From students’ PE exams</td><td>Quadratic function</td></tr>
<tr><td>T9</td><td>Instructor, organiser and assessor</td><td>Learner-interaction</td><td>Using the sprinkler as the origin, take the plane containing the jet of water as the $x-y$(XOY) plane. Set up a Cartesian coordinate system such that the line joining the origin to the point where the water lands forms the x-axis. This gives three points: $(0,5)$, $(30,10.2)$, and $(50,13.6)$ (units: cm). Find the distance from the sprinkler to the landing point of the water jet.</td><td>Procedures without connections</td><td>Close-ended</td><td>Societal</td><td>Personal experience

From a lawn sprinkler on campus</td><td>Quadratic function</td></tr>
</table>

			以浇水器为坐标原点，喷出的水柱所在平面为 xoy 平面，连接原点与水柱落地点的直线为 x 轴建立平面直角坐标系，得到三个点（0，5），（30，10.2），（50，13.6）单位是 cm，求水柱落地点离浇水器的距离。					
T10	Guide and instructor	Learner-interaction and content-performance	Given a cube with an edge length of 1 cm and a mass of 1 g, divide it evenly several times. Show how the number of cubes, the total surface area (cm^2) and specific surface area (m^2/g) change as the number of divisions increases. Describe the trends. Derive an algebraic expression for the case where the edge length is divided into n equal parts.<br><br>给定一个长为 1cm，重量为 1g 的正方体，进行数次平均分割.根据表格数据描绘出随着分割次数增多，正方体数量、总表面积、比表面积的变化情况。描述变化趋势。预测棱长被 n 等分割后的代数表达式。	Doing mathematics	Close-ended	Scientific	Social background<br><br>Xianmian (very thin noodles) from social media	Surface area & volume, proportional reasoning

<table>
<tr>
<td></td>
<td></td>
<td></td>
<td>
<table>
<tr><td>Times of Divisions</td><td>1</td><td>2</td><td>3</td><td>4</td><td>...</td><td>n</td></tr>
<tr><td>Edge Length of Cube(s) (cm)</td><td>1</td><td>$\frac{1}{2}$</td><td>$\frac{1}{3}$</td><td>$\frac{1}{4}$</td><td>...</td><td>$\frac{1}{n}$</td></tr>
<tr><td>Number of Cube(s)</td><td>1</td><td>8</td><td>27</td><td>64</td><td>...</td><td>n^3</td></tr>
<tr><td>Total Surface Area of Cube(s) (cm^2)</td><td>6</td><td>12</td><td>18</td><td>24</td><td>...</td><td>$6n$</td></tr>
<tr><td>Specific Surface Area of Cube(s) (m^2/g)</td><td>0.0006</td><td>0.0012</td><td>0.0018</td><td>0.0024</td><td>...</td><td>$0.0006n$</td></tr>
</table>
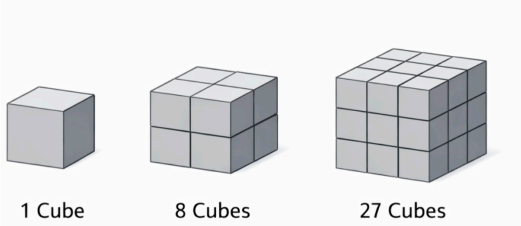

</td>
<td></td>
<td></td>
<td></td>
<td></td>
<td></td>
</tr>
<tr>
<td>T11</td>
<td>Guide</td>
<td>Learner-interaction</td>
<td>Analyse the data collected after planting chilli peppers, and try to explain the possible reasons behind the results.

分析种植辣椒后的数据，并试图解释可能的原因。</td>
<td>Procedures with connections</td>
<td>Open-ended</td>
<td>Occupational</td>
<td>Personal experience

From hands-on experience in growing chili peppers.</td>
<td>Basic arithmetic operations; statistical charts</td>
</tr>
</table>

<table>
<tr><td></td><td></td><td></td><td>Chilli Pepper Growth Change Log

Member(s): Recorded by:
<table>
<tr><td rowspan="2">Weeks</td><td colspan="2">Germination stage</td><td colspan="2">Seedling stage</td><td>Flowering and fruiting stage</td></tr>
<tr><td>Number of seeds sown</td><td>Number of seeds germinated</td><td>Minimum chilli plant height (mm)</td><td>Maximum chilli plant height (mm)</td><td>Yield (kg)</td></tr>
<tr><td>Week 1</td><td></td><td></td><td></td><td></td><td></td></tr>
<tr><td>Week 2</td><td></td><td></td><td></td><td></td><td></td></tr>
<tr><td>Week 3</td><td></td><td></td><td></td><td></td><td></td></tr>
</table></td><td></td><td></td><td></td><td></td><td></td></tr>
<tr><td>T12</td><td>Supporter</td><td>Learner-interaction</td><td>Design a car park on two existing vacant plots. (Plot 1: a rectangular plot measuring 30 m by 16 m; Plot 2: a square plot with sides of 40 m. There is a large tree in the middle; the thickest part of the trunk is 2 m in diameter.)

在已有的两块空地上，设计停车场。(第一块地：长方形，长 30 米，宽 16 米；第二块地：正方形，边长 40 米。中间有一棵大树，大树最粗部位为 2 米）</td><td>Doing mathematics</td><td>Open-ended</td><td>Occupational</td><td>Social background

From the car park in society</td><td>Rectangle, square, and basic arithmetic operations.</td></tr>
<tr><td>T13</td><td>Guide and supporter</td><td>Learner-interaction</td><td>Based on the imagery in Three Hundred Tang Poems, express your findings using statistical measures.</td><td>Doing mathematics</td><td>Open-ended</td><td>Scientific</td><td>Textbook

From</td><td>Sampling, statistical measure, statistical chart</td></tr>
</table>

			根据《唐诗三百首》中的意向，应用统计量表达你的发现。				supplementary materials in the Chinese language textbook	
T14	Guide	Learner-interaction	A rectangular athletic field measures 105 meters in length and 68 meters in width. Each sprinkler can irrigate a circular area with a radius of 10 meters. Design an arrangement of sprinklers that ensures the entire lawn is covered. Specify a reasonable number of sprinklers and their positions.<br><br>在一个长 105 米、宽 68 米的矩形田径场草坪中，已知每个浇水器可以覆盖半径为 10 米的圆形区域。请问如何布置浇水器才能覆盖整个草坪，并给出合理的数量和位置安排。	Doing mathematics	Open-ended	Personal	Personal experience<br><br>From the field in the campus	Rectangle and circle areas, coverage arrangement of circles

Interview protocol

Greetings:

First of all, thank you very much for taking the time to participate in this research. My name is XXX. This study focuses on the teaching and research of "project-based learning", with particular attention to mathematical problem solving within this context.

According to the Compulsory Education Curriculum Standards, "project-based learning mainly involves solving real-world problems using mathematical methods", and "the problems involved in project-based learning are mainly open-ended problems from the real world; problem solving requires transforming real-world problems into mathematical problems..."

In this study, **problem solving** refers to the process of addressing "challenging, non-routine problems for which students do not have a clear or immediate understanding of the ideas and approaches needed to reach a solution."

Today, I would mainly like to ask you about your personal views and opinions on "project-based learning". There are no right or wrong answers, nor are any views considered better or worse than others.

This interview will be audio-recorded. All recordings will be used solely for educational research purposes and will not be shared with anyone other than you and me. All statements made by you and by others will be anonymised, so please feel assured.

1. What is your length of teaching experience? What is your professional rank or level?

2. How would you describe the academic performance of the students you teach in comparison with other students in the district? How is their performance distributed?

3. In the process of designing project-based learning activities, do you usually work independently, or do you communicate and collaborate with colleagues? If there is communication and collaboration, what does it mainly involve? How does it support curriculum design and implementation? How is this similar to or different from your regular instructional design?

4. Problem solving in regular teaching appears in various types of lessons, such as new-content lessons, review lessons, and exercise-based lessons. How do you think problem solving in project-based learning should be positioned within the mathematics curriculum? In other words, what is the relationship between

problem solving in project-based learning and other areas of mathematics teaching?

5. How do you select the themes or content for project-based learning? How do you design the problem-solving tasks within it?

6. In the project-based learning activities you have implemented, how would you evaluate the achievement of cognitive objectives related to problem solving, such as knowledge and skills, processes and methods, as well as non-cognitive objectives, such as emotions, attitudes, and values? Please provide an overall evaluation.

7. Based on your experience, how does problem solving in project-based learning promote students' learning? Are there any aspects that have not yet met your expectations, or any shortcomings?

8. How do you understand the teacher's role in teaching problem solving within project-based learning? What about the students' role? Are these roles different from those in regular classroom teaching? If so, in what ways do they differ?

9-1. Regarding problem solving in project-based learning, which of the following statements do you agree with more? Please explain your view.

9-2. Regarding problem solving in regular teaching, apart from project-based learning, which of the following statements do you agree with more? Please explain your view.

There are no right or wrong answers, nor are any of these views considered better or worse than others.

A. You provide clues about the correct method or formula to solve the problem.

B. In addition to helping your students, you also ensure that they understand what they have written.

C. Without providing clues, you encourage your students to express their own ideas for solving the problem.

10. After implementing project-based learning, have your views or opinions about project-based learning changed? If so, what changes have occurred? What factors or events may have influenced your views or opinions about project-based learning?

11. What connections and implications do you think the implementation of problem solving in project-based learning has for teachers' instruction and students' learning in regular classroom teaching?

12. What factors do you think currently facilitate or hinder the implementation of problem solving in project-based learning?
